# Low Pulvinar Intensity in Susceptibility-Weighted Imaging May Suggest Cognitive Worsening After Deep Brain Stimulation Therapy in Patients With Parkinson's Disease

**DOI:** 10.3389/fneur.2019.01158

**Published:** 2019-10-31

**Authors:** Keita Matsuura, Masayuki Maeda, Masayuki Satoh, Ken-ichi Tabei, Tomohiro Araki, Maki Umino, Hiroyuki Kajikawa, Naoko Nakamura, Hidekazu Tomimoto

**Affiliations:** ^1^Department of Neurology, Graduate School of Medicine, Mie University, Tsu, Japan; ^2^Department of Neurology, Suzuka Kaisei Hospital, Suzuka, Japan; ^3^Department of Advanced Diagnostic Imaging, Graduate School of Medicine, Mie University, Tsu, Japan; ^4^Dementia Prevention and Therapeutics, Mie University, Tsu, Japan; ^5^Department of Neurosurgery, Suzuka Kaisei Hospital, Suzuka, Japan; ^6^Department of Radiology, Graduate School of Medicine, Mie University, Tsu, Japan

**Keywords:** pulvinar nuclei, susceptibility-weighted imaging, diffusion-weighted imaging, Parkinson's disease, deep brain stimulation, cognitive function

## Abstract

**Purpose:** Deep brain stimulation (DBS) is an established therapy for Parkinson's disease (PD). However, deteriorating cognitive function after DBS is a considerable problem for affected patients. This study was undertaken to assess whether pulvinar findings in susceptibility-weighted imaging (SWI) can suggest cognitive worsening.

**Methods:** We examined 21 patients with PD who underwent DBS along with SWI and neuromelanin-sensitive MR imaging (NMI). We further assessed pulvinar hypointensity based on the SWI findings and also the area of the substantia nigra (SN) pars compacta in NMI. We then examined associations among cognitive changes, pulvinar hypointensity, and SN area. The cognitive function of the patient immediately before surgery was compared with function at 1 year postoperatively.

**Results:** Pulvinar hypointensity in SWI was found in 11 of 21 patients with PD at baseline. One year postoperatively, six of the 21 patients demonstrated a Mini-Mental State Examination score that was ≥3 points lower than the baseline score. We observed pulvinar hypointensity in SWI before DBS surgery in five of these six patients (*p* = 0.072). During the first postoperative year, six of 21 patients reported both transient or permanent hallucinations; we observed pulvinar hypointensity in these six patients, while 10 patients without pulvinar hypointensity had no hallucinations.

**Conclusion:** Pulvinar hypointensity in SWI in patients with PD may provide information that is useful for suggesting cognitive deterioration after DBS treatment.

## Introduction

Deep brain stimulation (DBS) is an established therapy for Parkinson's disease (PD) (1). However, patients who undergo DBS may experience side effects such as headaches, seizures, recall difficulty, and postoperative deterioration of cognitive function (2). Frontal 18-fluorodeoxyglucose positron emission tomography (PET) activity is reportedly related to cognitive outcome after DBS of the subthalamic nucleus (STN) in patients with advanced PD disease (3). In daily practice, however, it is difficult to use PET scans.

Recently, hypointensity of the pulvinar nucleus on fluid-attenuated inversion recovery (FLAIR) images has been found in patients with Alzheimer's disease; this is suspected to represent abnormal iron accumulation (4). We have shown that a low signal from the pulvinar nucleus on diffusion-weighted imaging (DWI) is associated with hallucinations in dementia patients (5). Furthermore, some reports have indicated a relationship between pulvinar change and Lewy body dementia (DLB) (6–8). In particular, Erskine et al. reported that α-synuclein was present throughout the pulvinar in DLB (6). Notably, α-synuclein can bind Fe(II) and Fe(III) (9–12), and susceptibility-weighted imaging (SWI) exploits the tissues' magnetic properties, such as blood or iron content (13). Hence, we hypothesized that hypointensity of the pulvinar nucleus in SWI will suggest cognitive worsening after DBS during prodromal cognitive impairment in patients with PD because it may represent α-synuclein pathology.

Furthermore, neuromelanin-sensitive MR imaging (NMI) is a useful tool for the diagnosis and follow-up of patients with PD (14–19). Hatano et al. reported that patients with PD with motor complications (MC) had markedly smaller substantia nigra (SN) pars compacta areas in NMI compared with patients with PD without MC (20). Several papers have reported a correlation between motor symptom severity and NMI signal changes in patients with PD (16–19). However, there have been no reports of correlations between cognitive function and NMI signal changes in patients with PD. In this study, we aimed to evaluate the relationships between cognitive worsening in patients with PD after DBS, the hypointensity of the pulvinar nucleus in SWI, and the SN area in NMI.

## Materials and Methods

This study was conducted retrospectively. Inclusion criteria were as follows: patients with PD who fulfilled the United Kingdom Brain Bank criteria (21) and underwent DBS therapy at our institution between November 2010 and April 2016. Twenty-one patients (16 women, five men; mean age: 62.1 years) matched these criteria. This study was approved by the Institutional Review Boards of our institutions, and informed consent was provided by all patients before enrollment in the study.

All patients underwent MRI at a 3T MRI unit (Verio; Siemens AG, Erlangen, Germany) with a 32-channel head coil. MR sequences consisted of SWI, DWI, three-dimensional (3D)-FLAIR, NMI, and 3D-T1-weighted imaging. The SWI parameters were as follows: repetition time (TR), 25 ms; echo time (TE), 20 ms; flip angle, 16°; field of view (FOV), 210 mm; matrix size, 510 × 512; section thickness, 1.2 mm; acquisition time, 3 min 42 s. The DWI parameters were as follows: TR, 5,900 ms; TE, 85 ms; FOV, 230 × 230 mm; matrix size, 114 × 114; slice thickness, 3.0 mm; acquisition time, 1 min 17 s. The 3D-FLAIR parameters were as follows: TR, 10,000 ms; TE, 617 ms; flip angle, T2 var; FOV, 267 × 267 mm; matrix size, 256 × 256; slice thickness, 1.1 mm; acquisition time, 6 min 20 s. The pulse sequence used for NMI was a T1-weighted fast spin-echo technique; TR, 550 ms; TE, 11 ms; echo train length, 4; FOV, 200 mm; matrix size, 448 × 311 (pixel size: 0.45 × 0.64 mm); slice thickness, 2.5 mm (gapless, 6-averaged, 12 slices); acquisition time, 9 min (14, 16, 18). For voxel-based morphometry (VBM), the parameters used for 3D-T1-weighted imaging were as follows: TR, shortest; TE, 15 ms; flip angle, 90°; FOV, 230 × 230 mm; matrix size, 256 × 256; slice thickness, 1.1 mm; acquisition time, 6 min 20 s.

All patients underwent bilateral electrode placement for STN (17 patients) or pallidal (four patients) DBS. Electrodes (Medtronic DBS lead models 3389 and 3387, Medtronic, Minneapolis, MN, USA; and the Vercise® DBS lead, Boston Scientific, Natick, MA, USA) were implanted under local anesthesia using a Leksell stereotactic frame (Elekta Instruments AB, Stockholm, Sweden) and anatomical (MRI and computed tomography) and physiological targeting. Based on microelectrode recordings, electrodes were considered correctly located in the target region. Impulse generators (Activa SC/RC, Medtronic; Vercise® system, Boston Scientific) were implanted and connected during a second surgical procedure on the same day.

The levodopa equivalent daily dose (LEDD) for each patient was calculated as follows: 100 mg L-dopa/decarboxylase inhibitor = 1 mg pramipexole = 5 mg ropinirole = 3.3 mg/day rotigotine = 4 mg cabergoline = 70 mg L-dopa/decarboxylase inhibitor with entacapone (22–24).

**Figure 1:**
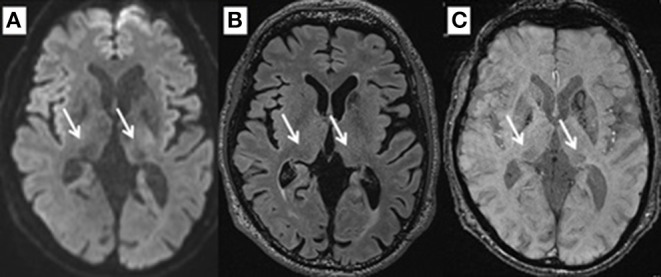
Pulvinar hypointensity MRI of a 61-year-old man in DWI **(A)**, FLAIR **(B)**, and SWI **(C)**.

By performing SWI, FLAIR, and DWI at the level of 2 mm above the anterior to posterior commissure (AC–PC) line, signal intensities for the pulvinar nucleus were evaluated for normal intensity or hypointensity by two reviewers in a blinded manner (Figure 1). The SN area was measured at the section through the inferior edge of the inferior colliculus using ImageJ (National Institutes of Health, Bethesda, MD, USA). Briefly, image files containing neuromelanin-related contrast (NRC) at the section through the inferior edge of the inferior colliculus were imported to ImageJ, converted into 8-bit files, and smoothed. Next, the SN threshold was adjusted to a level that eliminated noise contrast, leaving NRC in the SN and additional contrast in two small areas lateral to the aqueduct. The areas filled with white were originally shown in red on the computer color display. The number of pixels in each area was calculated automatically (16, 18, 19).

We also performed N-isopropyl-p-[123I]-iodoamphetamine ([123I]-IMP) single-photon emission computerized tomography (SPECT) scans for all patients. All subjects were injected with 167 MBq of [123I]-IMP while in a supine resting state with their eyes closed. After 5 min, brain SPECT scanning was performed for 25–30 min using an E.CAM system, an LMEGP collimator, and a GMS-5000 WorkStation (Toshiba, Tokyo, Japan). Axial images were obtained by filtered back-projection methods. Cerebral blood flow (CBF) was measured by Graph Plot Analysis (25).

MRI data for VBM were analyzed using SPM12 (Wellcome Institute of Neurology, University College London, London, UK) running on MATLAB R2012a (MathWorks, Natick, MA, USA). In the pre-processing phase, images were set to match the AC-PC line using an automated MATLAB script. The images were then visually inspected to detect possible scan issues such as field distortion and movement artifacts. Reoriented images were corrected for intensity inhomogeneity and segmented into gray matter (GM), white matter (WM), cerebrospinal fluid, and other tissues outside of the brain by SPM12 tissue probability maps. The images were registered with the East Asian Brains International Consortium for Brain Mapping space template through affine regularization. We created a population-specific template using the SPM12 DARTEL template procedure to compare groups directly, with or without pulvinar hypointensity in SWI, cognitive worsening, and hallucination; thus, we investigated whole-brain GM differences between groups. GM and WM segments were inputted into high-dimensional DARTEL to create non-linear, modulated-normalized, GM images that were smoothed using a Gaussian kernel of 8 mm full width at half maximum. No participants were excluded from analysis after these steps.

For the region of interest of pulvinar nucleus analyses, we assessed the statistical significance at a voxel threshold of *p* < 0.005 (uncorrected); contiguous clusters of at least 10 voxels were reported. We obtained both Montreal Neurological Institute (MNI) and Talairach coordinates to detect the anatomical regions of the clusters. We used a transformation from Matthew Brett (http://imaging.mrc-cbu.cam.ac.uk/imaging/MniTalairach) to convert MNI coordinates to Talairach coordinates, and Talairach Client 2.4.3 was used to identify the anatomical regions corresponding to Talairach coordinates (26). We performed assessments including the Unified Parkinson's Disease Rating Scale (UPDRS), LEDD, Mini-Mental State Examination (MMSE), Frontal Assessment Battery (FAB), the Trail Making Test (TMT), and the Center for Epidemiologic Studies Depression Scale (CES-D) for all patients before DBS (i.e., baseline) and at 1 year postoperatively (27–31). We also checked for the presence or absence of hallucinations based on medical records.

All statistical analyses were performed using SPSS software (version 23, IBM Corp., Armonk, NY, USA). To compare the Hoehn-Yahr stage, UPDRS, use of dopamine agonist, entacapone, selegiline and zonisamide, MMSE, FAB, and CES-D between baseline and 1 year postoperatively, we used Wilcoxon signed-rank tests. To compare the LEDD, L-dopa dosage, and TMT-A between baseline and 1 year postoperatively, we used paired *t*-tests. To compare the MMSE score change, MMSE score at baseline, and Hoehn-Yahr stage between the groups with a worsened and with a stationary or better MMSE score, we used Mann–Whitney's *U*-test. To compare age, disease duration, and LEDD between the groups with a worsened and with a stationary or better MMSE score, we used unpaired *t*-tests. To compare the change in TMT-A and FAB scores between the groups with pulvinar isointensity and pulvinar hypointensity in SWI, we used Mann-Whitney's *U*-test. Fisher's exact test was used to analyze pulvinar hypointensity or isointensity in the SWI, DWI, and FLAIR groups and to compare between groups with SN areas ≥12 and <12 for changes in MMSE scores. Correlations between changes in the MMSE score and SN area, as well as between UPDRS and SN area, were analyzed using Spearman's rank correlation coefficient. We used a Student's *t*-test to compare CBF between groups. An α level of 0.05 was considered statistically significant.

## Results

At the time of the DBS operation, the patients' average age (mean ± standard deviation) was 62.1 ± 8.6 years. Immediately after DBS, the average motor performance (Hoehn-Yahr stage, UPDRS) significantly improved, and LEDD and L-dopa dosage significantly decreased (Table 1). Cognitive performance (MMSE, FAB, and TMT-A) and the depression scale remained unchanged (Table 1). One year postoperatively, the average Hoehn-Yahr stages in on and off states, respectively, improved compared with baseline (*p* = 0.031 and *p* < 0.001, respectively) (Table 1). All patients had no hallucinations at the baseline.

**Table 1:** Patient profile.

	**Pre-operation**	**After 1 year**
Age	62.1 ± 8.6	
Sex (M:F)	5:16	
Disease duration (years)	11.3 ± 6.3	
DBS target	STN17 GPi4	
Hoehn-Yahr stage (on state)	2.4 ± 0.8	2.1 ± 0.9^*^
Hoehn-Yahr stage (off state)	3.7 ± 0.8	2.7 ± 0.8^**^
UPDRS Part I	3.0 ± 2.6	0.95 ± 1.28^**^
UPDRS Part II	9.5 ± 6.2	8.0 ± 5.9
UPDRS Part III	18.3 ± 12.3	11.4 ± 8.5^**^
UPDRS Part IV	7.4 ± 2.9	3.3 ± 2.7^**^
LEDD (mg)	694 ± 294	427 ± 219^**^
L-Dopa (mg)	376 ± 161	208 ± 121^**^
DA (use rate; %)	86%	81%
Entacapone (use rate; %)	71%	52%
Selegiline (use rate; %)	24%	24%
Zonisamide (use rate; %)	33%	38%
MMSE	26.24 ± 3.38	25.86 ± 5.14
FAB	13.76 ± 3.08	14.75 ± 2.40
TMT-A (s)	200 ± 149	153 ± 81
CES-D	16.4 ± 13.0	13.2 ± 13.3

* *p < 0.05*,

** *p < 0.01. DBS, deep brain stimulation; STN, subthalamic nucleus; GPi, internal segment of globus pallidus; UPDRS, the Unified Parkinson's disease rating scale; LEDD, levodopa equivalent daily dose; DA, dopamine agonist; MMSE, Mini-Mental State Examination; FAB, Frontal Assessment Battery; TMT, Trail Making Test; CES-D, the Center for Epidemiologic Studies Depression Scale*.

**Figure 2:**
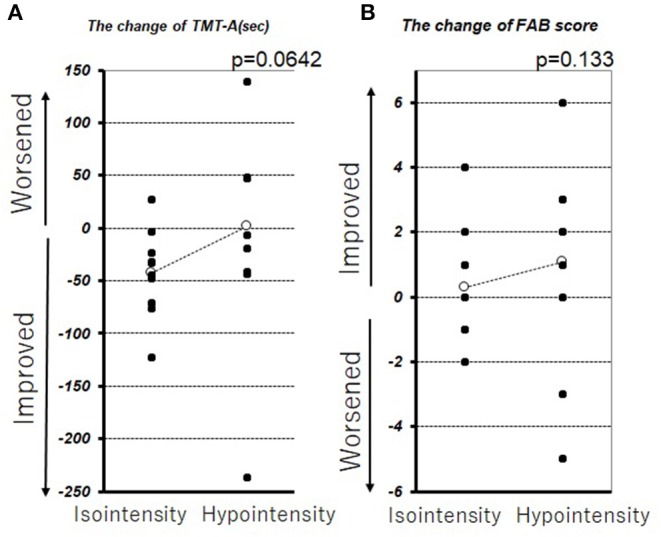
Change in TMT-A **(A)** and FAB **(B)** before DBS therapy and 1 year after DBS therapy. The white circle represents the average.

In this series, pulvinar hypointensity, as assessed by visual inspection by performing SWI, DWI, and FLAIR, was present in 11, 9, and 7 patients, respectively (Figure 1). One year postoperatively, six of 21 patients had MMSE scores < 24. Four of these six cases showed a reduction of ≥3 points in the MMSE score from baseline, and five of the six cases experienced transient or permanent hallucinations. The type of hallucination was both visual and auditory in three cases and only visual in three cases. We observed pulvinar hypointensity in SWI in all six patients (Table 2, *p* = 0.0057). Six of 21 patients showed a reduction of ≥3 points in the MMSE score from baseline; five of these six patients showed pulvinar hypointensity in SWI (Table 2, *p* = 0.072). During the observation period, six of 21 patients reported both transient and permanent hallucinations; all six patients showed hypointensity in SWI. In contrast, 10 patients without pulvinar nucleus hypointensity in SWI reported no hallucinations (Table 2, *p* = 0.0057). We compared age, disease duration, LEDD, and MMSE score at baseline between the groups with a worsened and with a stationary or better MMSE score 1 year postoperatively. A difference was observed between age at the operation and pulvinar hypointensity in SWI (*p* = 0.100 and 0.072, respectively; Table 3). Nine cases showed pulvinar hypointensity in DWI and no association with the MMSE score, deterioration of cognitive function, or occurrence of hallucinations 1 year postoperatively (*p* = 0.48, 0.93, and 0.79, respectively). Seven cases showed pulvinar hypointensity by FLAIR and no association with the MMSE score, deterioration of cognitive function, or occurrence of hallucinations 1 year postoperatively (*p* = 0.15, 0.66, and 0.30, respectively). No significant differences were observed in the change in TMT A time between the groups with pulvinar isointensity (−42.8 ± 41.3) and hypointensity (2.2 ± 114.7, *p* = 0.0642) in SWI. There was no significant difference in the change in FAB score between the groups with pulvinar isointensity (0.3 ± 1.8) and hypointensity (1.1 ± 3.1, *p* = 0.133) in SWI (Figure 2). The group with pulvinar hypointensity in SWI was divided into two. One was a group with an MMSE score reduction of ≥3 points, and the other was a group with an MMSE score reduction of <3 points. Age, disease duration, LEDD, and severity (Hoehn-Yahr stage) at the baseline were not significantly different between the two groups. The intraclass correlation coefficient of visual inspection of the iso- or hypointensity of the pulvinar nucleus in SWI was 0.931, indicating excellent correlation.

**Table 2:** Relationship between cognitive function and pulvinar hypointensity in SWI.

	**MMSE score 1 year after** ** DBS operation**		**MMSE score worsened over** ** 3 points from baseline**		**Hallucination occurred** ** during follow-up period**	
**Pulvinar in SWI**	**Normal**	**Under 24**	**Stationary or better**	**Worsened**	**Nothing**	**Occurred**
Hypointensity	5	6	6	5	5	6
Isointensity	10	0	9	1	10	0
		*p* = 0.0057		*p* = 0.072		*p* = 0.0057

**Table 3:** Comparison between the worsened and stationary or better MMSE score groups.

	**MMSE score worsened over 3 points from baseline**		
	**Worsened (6 cases)**	**Stationary or better** ** (15 cases)**	***p*-value**
MMSE score pre-operation	26.3 ± 2.4	26.2 ± 3.8	0.937
Change in MMSE score	−6.0 ± 3.9	1.9 ± 2.3	0.00110
Age (years)	67.0 ± 6.5	60.1 ± 8.8	0.100
Disease duration (years)	10.3 ± 6.3	11.7 ± 6.7	0.666
LEDD (mg)	631 ± 410	719 ± 258	0.559
Hoehn-Yahr stage (On/Off)	3.0 ± 0.6/3.7 ± 0.8	2.2 ± 0.8/3.7 ± 0.9	0.075/0.577
Pulvinar hypointensity in SWI	83% (5/6)	40% (6/15)	0.072

*MMSE, Mini-Mental State Examination; LEDD, levodopa equivalent daily dose; SWI, susceptibility-weighted imaging*.

**Figure 3:**
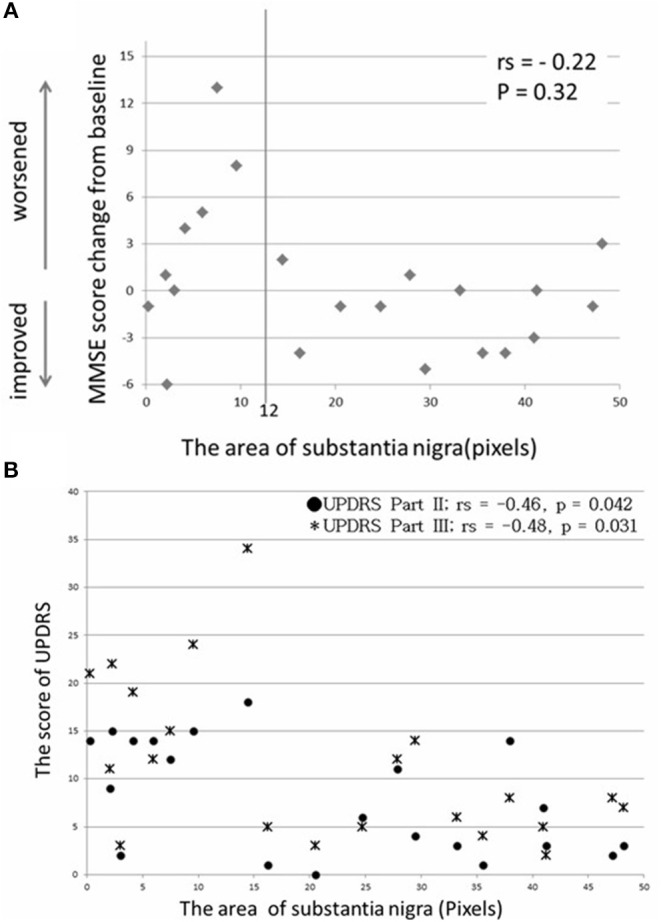
No significant correlation was found between SN area and MMSE score change **(A)**, and a moderate correlation was found between SN area and UPDRS score 1 year postoperatively **(B)**.

No correlation was found between the SN area calculated by NMI and the MMSE score change (rs = −0.22, *p* = 0.32) (Figure 3A). However, in the group with ≥12 pixels of the SN area (13 patients), only one patient showed an MMSE score reduction of 3 points between baseline and 1 year postoperatively (*p* = 0.0046) (Table 4). Moreover, the SN area in 21 patients with PD by NMI was correlated with UPDRS parts II and III, when assessed in the on state 1 year postoperatively (rs = −0.46 and −0.48, *p* = 0.042 and 0.031) (Figure 3B).

**Table 4:** Relationship between cognitive function and the SN area in NMI.

**SN area (pixels)**	**MMSE score**	
	**Stationary or better**	**Worsened**
≥12	12	1
<12	4	4
		*p* = 0.0018

**Figure 4:**
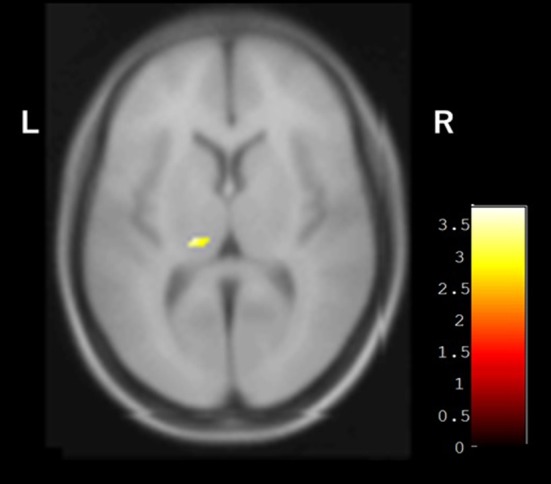
VBM analysis showing the difference between groups with and without pulvinar hypointensity in SWI. Left pulvinar volumes of the group with pulvinar hypointensity in SWI were smaller than those of the group without pulvinar hypointensity in SWI.

In the baseline SPECT evaluation, the average occipital CBF in the group with hallucinations (30.66 ± 3.27 ml/100 g/min) was significantly lower than that of the group without hallucinations (38.90 ± 6.30 ml/100 g/min, *p* = 0.0036). In the VBM analysis, the volume of the left pulvinar nucleus with isointensity in SWI was greater than that of the pulvinar nucleus with hypointensity in SWI (Figure 4). On the other hand, there was no difference between these two groups in other areas, including the caudate nucleus, periventricular white matter area, and centrum semiovale white matter area.

## Discussion

In the present study, we found that pulvinar hypointensity in SWI helps suggest cognitive worsening and the emergence of hallucinations. The SN area seen on NMI suggests the score of UPDRS part II and III 1 year after DBS surgery. In general, the cognitive impairment of patients with PD is strongly related to their quality of life (32). Therefore, patients with PD need to avoid deterioration of cognitive function and hallucinations induced by DBS surgery. STN-DBS therapy is beneficial for some elderly patients with PD aged ≥70 years; however, their clinical benefits are inferior to those of younger patients (33). Indeed, elderly patients with PD aged ≥70 years are frequently excluded from DBS therapy worldwide (34, 35). This study also suggested MMSE deterioration with age.

The present study suggested a correlation between pulvinar hypointensity in SWI and MMSE score change after DBS. Recently, it was reported that the pulvinar nucleus is involved in visual attention and modulation of behavioral responses through indirect cortico-cortical connections (36). A neuroimaging study demonstrated modulation of responses in the pulvinar nucleus with the usage of selective attention tasks that direct attention to a particular spatial location, shift attention across the visual fields, or exclude unwanted information (36). We previously reported a correlation between pulvinar hypointensity in DWI and hallucination in dementia patients (5), which is probably due to functional changes in lateral and inferior pulvinar subnuclei related to their strong connections with the visual cortex (36, 37). Moon et al. reported that the pulvinar nucleus exhibited a low signal on FLAIR images in Alzheimer's disease, describing potential iron accumulation on T2-weighted images (4). Based on these reports, pulvinar hypointensity in SWI may reflect iron deposition in the pulvinar nucleus. However, we did not observe significant correlations between pulvinar hypointensity on DWI or FLAIR and the MMSE score. Pulvinar hypointensity was detected by FLAIR, DWI, and SWI in 7, 9, and 11 cases, respectively. One possible explanation of the lack of correlation with MMSE score in DWI and FLAIR is that the detectability for pulvinar hypointensity was lower in these methods than in SWI. In this regard, further studies including a larger number of cases will be needed. There was no significant difference in age, disease duration, LEDD, and severity between the worsened-cognition group and the stationary or better group. We do not know why these differences occur, and further investigations are needed.

Recently, a relationship was reported between changes in the pulvinar nucleus and the presence of DLB, supported by MRI and pathological findings (8, 9). Accumulation of α-synuclein in the pulvinar nucleus has been demonstrated in pathological analysis of DLB patients (8). Furthermore, cultured neurons over-expressing α-synuclein exhibited iron accumulation when exposed to excess iron (38). Based on these findings, the low signal in SWI may reflect the spread of α-synuclein, leading to cognitive impairment and hallucination. In prior findings and the present results, pulvinar hypointensity in SWI may be compatible with stages 5 and 6 of the Braak hypothesis of Lewy body pathology in patients with PD (39). The smaller left pulvinar nucleus in the group with pulvinar hypointensity observed during VBM analysis may reflect the change in pulvinar nuclei and support this hypothesis (Figure 4). Moreover, Watson et al. reported that the region of the left pulvinar and ventral lateral nucleus was associated with impaired attentional function in DLB (7). In a comparison between pulvinar hypointensity and isointensity in SWI, the TMT A of isointensity group showed improvement after surgery, whereas no change was observed in the hypointensity group. The result of TMT-A did not reach a significant level because of an outlier in the hypointensity group, which showed remarkable improvement (Figure 2). Therefore, TMT-A might improve after surgery in the pulvinar isointensity group and worsen in the hypointensity group. The fact that there was atrophy of the left pulvinar nuclei of the hypointensity group in the significant hemisphere is congruent with the fact that pulvinar nuclei are related to visual attention (36, 37). It was suggested that, because of visual attention deficit, the result of TMT-A in the hypointensity group tended to worsen after surgery. Regarding the result of FAB, there was also no difference between the pulvinar isointensity and hypointensity groups. Though FAB is regarded as one of the tests for frontal function, it includes various tasks such as similarities, motor series, and Go–No Go, not necessarily just attention (29). It can reasonably be concluded that diversity of FAB can occur with no tendency in the hypointensity group.

Conversely, there was no direct association between the SN area in NMI and the MMSE score, and only one patient showed considerable deterioration (≥12 pixels). Moreover, 1 year postoperatively, there was a correlation between the SN area in NMI and UPDRS parts II and III. Thus, a larger SN area in NMI indicated possible motor function improvement. Cognitive function is suggested not to worsen when the SN area maintains a specific level of positive pixels. However, a direct association between the SN area and cognitive function remains unclear. Therefore, this should be examined in a prospective study with a larger number of patients.

The present study had several limitations. First, pulvinar hypointensity was assessed based on visual inspection. Future studies should confirm and extend our findings using a quantitative method, such as quantitative susceptibility mapping (QSM). We are currently conducting the study using QSM. Second, in this study, we could not distinguish and analyze changes in specific pulvinar subnuclei. The pulvinar nucleus has several connections, and there are differences among subnuclei. If pulvinar subnucleus changes can be elucidated, such information may be more useful for clarifying the pathophysiological significance of this phenomenon.

Pulvinar hypointensity observed in SWI in patients with PD before DBS treatment reflects the cognitive function after DBS, and the SN area on NMI reflects the UPDRS parts II and III at 1 year after DBS, which may provide useful information regarding the prognosis of cognitive and motor functions after DBS.

## Data Availability Statement

The data are not available for public access because of patient privacy concerns but are available from the corresponding author on reasonable request.

## Ethics Statement

The studies involving human participants were reviewed and approved by Mie University Hospital ethics committee and Suzuka Kaisei Hospital ethics committee. Written informed consent for participation was not required for this study in accordance with the national legislation and the institutional requirements.

## Author Contributions

KM, TA, MM, and HT planned the study. KM, HK, and NN assessed patients. KT, MU, and KM analyzed the data. KM, MM, MS, KT, and HT wrote and corrected the manuscript.

### Conflict of Interest

The authors declare that the research was conducted in the absence of any commercial or financial relationships that could be construed as a potential conflict of interest.
